# Awareness of obstetricians for long-term risks in women with a history of preeclampsia or HELLP syndrome

**DOI:** 10.1007/s00404-021-06181-w

**Published:** 2021-08-18

**Authors:** Pilar Palmrich, Carina Binder, Harald Zeisler, Bettina Kroyer, Petra Pateisky, Julia Binder

**Affiliations:** 1 Department of Obstetrics and Feto-Maternal Medicine, Medical University of Vienna, Vienna, Austria; 2 Department of Pathology, Medical University of Vienna, Vienna, Austria; 3 Center for Medical Statistics, Informatics and Intelligent Systems, Medical University of Vienna, Vienna, Austria

**Keywords:** Pregnancy, Preeclampsia, Hypertensive disorders of pregnancy, Cardiovascular disease

## Abstract

**Purpose:**

Hypertensive disorders of pregnancy are still a leading cause of maternal and neonatal morbidity and mortality worldwide. Women with a history of preeclampsia have an increased risk for future cardiovascular and cerebrovascular disease, renal disease as well as diabetes mellitus. There is little knowledge on postpartum risk management. The aim of this study was to assess follow-up care for patients after pre-eclampsia or HELLP syndrome.

**Methods:**

This questionnaire-based cross-sectional study aimed to evaluate the current recommendations of obstetricians in Austria regarding follow-up care, long-term risk counselling and risk of recurrence in future pregnancies after preeclampsia or HELLP syndrome. Data were collected using a survey, based on recommendations given by three substantial guidelines on hypertensive disorders of pregnancy, which was distributed via e-mail to 69 public obstetric departments in Austria. Each obstetric department was required to answer one questionnaire per local protocol.

**Results:**

Our results revealed that of the 48 participating hospitals most obstetricians are aware of the importance of follow-up care for women after a pregnancy complicated by preeclampsia. Our data show that most physicians counselled patients about the future cardiovascular health risks associated with preeclampsia or HELLP syndrome (79.2%). Most obstetricians recommended lifestyle modification (77.1%) and continued blood pressure measurements (97.9%). All centers stated to counsel about the risk of recurrence (100%). However, counselling regarding follow-up care to exclude kidney damage (37.5%) and underlying diseases like thrombophilia (39.6%) were less prioritized.

**Conclusions:**

We were able to show that counselling concerning the risk of long-term cardiovascular disease and risk of recurrence after a pregnancy complicated by preeclampsia or HELLP syndrome has been established in obstetric departments in public hospitals. Regarding the evaluation of underlying chronic diseases such as thrombophilia or renal disease, as well as counselling on the future risk of renal disease is still improvable according to our data. Further evaluation of follow-up care after hypertensive disorders of pregnancy in the outpatient and private sector and implementation of structured guidelines for follow-up, as well as screening for cardiovascular disease are necessary to ensure adequate risk management and to provide opportunities for prevention.

**Supplementary Information:**

The online version contains supplementary material available at 10.1007/s00404-021-06181-w.

## Introduction

Cardiovascular disease (CVD) is the most prevalent cause of death in women worldwide [1]. Hypertensive disorders of pregnancy (HDP), such as preeclampsia (PE), are known to be contributing factors to the risk of CVD [2, 3], affecting 5–10% of pregnancies in the developed world [4]. HDP have been the leading cause of maternal and neonatal morbidity and mortality worldwide in the last decades. PE is a pregnancy-specific disorder defined by new-onset hypertension during pregnancy and at least one additional new-onset organ manifestation including proteinuria, thrombocytopenia, increased liver enzymes, neurological symptoms such as heachaches with visual disturbances, right upper quadrant abdominal pain and/or placental insufficiency manifesting in intrauterine growth restriction. Superimposed preeclampsia is defined as the new onset of one or more of the above features of preeclampsia occurring in addition to chronic hypertension during pregnancy [5]. Hemolysis, elevated liver enzymes and low platelets syndrome (HELLP syndrome) is a pregnancy-specific disorder characterized by increased transaminases, reduced platelet counts and hemolysis [5]. The underlying pathophysiology and pathomechanisms leading to PE are not yet fully understood. Multiple pathogenetic mechanisms have been suggested in this disorder, however, the common hypothesis implicats an underlying imbalance between angiogenic and antiangiogenic factors deriving from placental malperfusion and oxidative stress as a result of abnormal placentation [6, 7] as well as more recently a cardiovascular origin of the disease was suggested [5–7]. Even though the clinical manifestation of PE commonly ceases within few days after delivery, studies on maternal hemodynamic changes in women with HDP revealed persistent long-term cardiac alterations of up to 2 years after PE and increased lifetime risk of essential hypertension, cardiovascular disease and stroke [8–10], disturbances in renal function [11] as well as increased risk of developing diabetes [12]. It has also been shown that women with a history of PE have an up to five- to 12-fold increased risk of developing end-stage renal disease later in life [11, 13]. The association between PE and future CVD is no surprise given the fact that several studies have revealed that women with PE are in a state of cardiac dysfunction [5, 14]. PE has been shown to be associated with cardiovascular changes such as a decrease in cardiac output, increased systemic vascular resistance and left ventricular diastolic dysfunction [5, 6, 14, 15]. Studies on maternal hemodynamic changes in women with PE revealed persistent long-term cardiac alterations of up to two years after PE and increased lifetime risk of essential hypertension and cardiovascular disease [9]. Long-term follow-up studies have revealed a particularly high lifetime risk to develop cardiovascular disease as well as significantly higher cardiovascular mortality in cases of early-onset PE compared to women with a history of late-onset PE [2, 8, 10]. Additionally, women with a history of HDP have an increased risk of recurrence, with reported recurrence rates of more than 20% [16]. The overwhelming evidence that the obstetric history, particularly history of HDP, offers a unique risk marker to identify young women at risk for future cardiovascular and renal disease, has resulted in the incorporation of PE and HELLP syndrome (Hemolysis Elevated Liver Enzymes Low Platelet Syndrome) as a female-specific risk factor in several guidelines, such as the 2011 American Heart Association guideline for prevention of CVD, the 2014 American Heart Association stroke prevention guideline and the 2016 European Society of Cardiology guideline CVD prevention [17–20]. Adequate screening and preventive measures, therefore, are key in this young population of women at risk of future cardiovascular and renal disease. However, information on the expertise of obstetricians and the quality of their counselling regarding postpartum management for patients after HDP is scarce. Current recommendations in Austria cover counselling on long-term risk for cardiovascular disease, risk of recurrence and lifestyle modifications as well as education on prevention in future pregnancies. Concerning follow-up after PE, the current guideline [21] recommends evaluation of secondary causes of hypertensive pregnancy complications at 3–6 months after delivery, including assessment of blood pressure, heart rate, BMI, urin status, laboratory assessments such as lipid profile and serum electrolytes, and assessment of kidney damage. Furthermore, recommendations include assessment of thrombophilia in severe cases and echocardiography in persistent hypertension. Currently, the recommendations regarding screening after PE include investigation of other cardiovascular risk factors and review of blood pressure, fasting glucose, BMI, and lipid status at regular intervals (at least every 5 years) [21]. Considering the significance of this issue, we aimed to assess the quality of follow-up care for patients after pre-eclampsia or HELLP syndrome.

## Material and methods

This study was designed as a cross-sectional study to evaluate the follow-up care for patients after PE or HELLP syndrome in Austria. The data were collected using a questionnaire-based survey. The questionnaire was distributed via e-mail to all 69 public obstetric departments in Austria. As the study was designed as a cross-sectional study only one questionnaire per obstetric department was required to be returned. Participants were selected by the head of each department and asked to answer according to their local protocol. Returned questionnaires were filled in anonymously.

### Questionnaire

The questionnaire was developed at the Department of Obstetrics and feto-maternal Medicine at the Medical University of Vienna. It was designed after research of current literature regarding follow-up care of patients affected by PE or HELLP syndrome based on guidelines from three different institutions, the National Institute for Health and Care Excellence, American College of Obstetrics and Gynaecologists and Arbeitsgemeinschaft der Wissenschaftlichen Medizinischen Fachgesellschaften in Germany [21–23]. The questionnaire consists of 17 questions divided into four sections covering sociodemographic data, long-term risk of cardiovascular disease and kidney damage after PE, diagnostics of underlying disorders such as antiphospholipid syndrome or systemic lupus erythematosus, and risk of recurrence. The primary outcome of the study was to evaluate the follow-up care for patients after PE or HELLP syndrome, specifically assessing counselling on long-term risk of cardiovascular and renal disease, risk of recurrence of HDP as well recommendations on follow-up care including recommendations on postpartum diagnostics of possible underlying disease.

### Comparison of the guidelines

In Table S1 we present the three guidelines [21–23] with regards to the questions in our survey. Questions 1–3 assessed age, gender and location of the clinic. Question 4 was eliminated to ensure the data privacy of the study participants.

### Statistical analysis

For descriptive purposes, a table analyzing the study participants’ county of origin (with reference to Austria) by gender (female, male, not mentioned) was generated, listing both the absolute and relative frequencies. Continuous variables were represented as median and interquartile range or mean and standard deviation depending on distribution assumptions. Categorical variables were represented as number and percentage of the total. Chi-squared test was used for group comparisons. For the number of recommendations, a *t*-test was fit to compare the medians, resulting in *p*-values, respectively. To illustrate the absolute and relative frequencies of the outcome tables were created for each question of the questionnaire.

## Results

The questionnaire was sent to all 69 public obstetrical departments of which 48 participated in the survey (69.6%). The median number of questions on possible recommendations answered with yes was 6 (IQR 3 [95% CI 5.85–6.39]) of 9 questions (70.6%). There was no difference in the median number of recommendations (median 6, IQR 3) between male and female participants (*p* = 0.690). Due to the small sample size, no statistically significant difference in dichotomised age, gender and region was found in regard to any of the sections of the questionnaire.

### Long-term risk of cardiovascular disease and renal disease

**Fig. 1:**
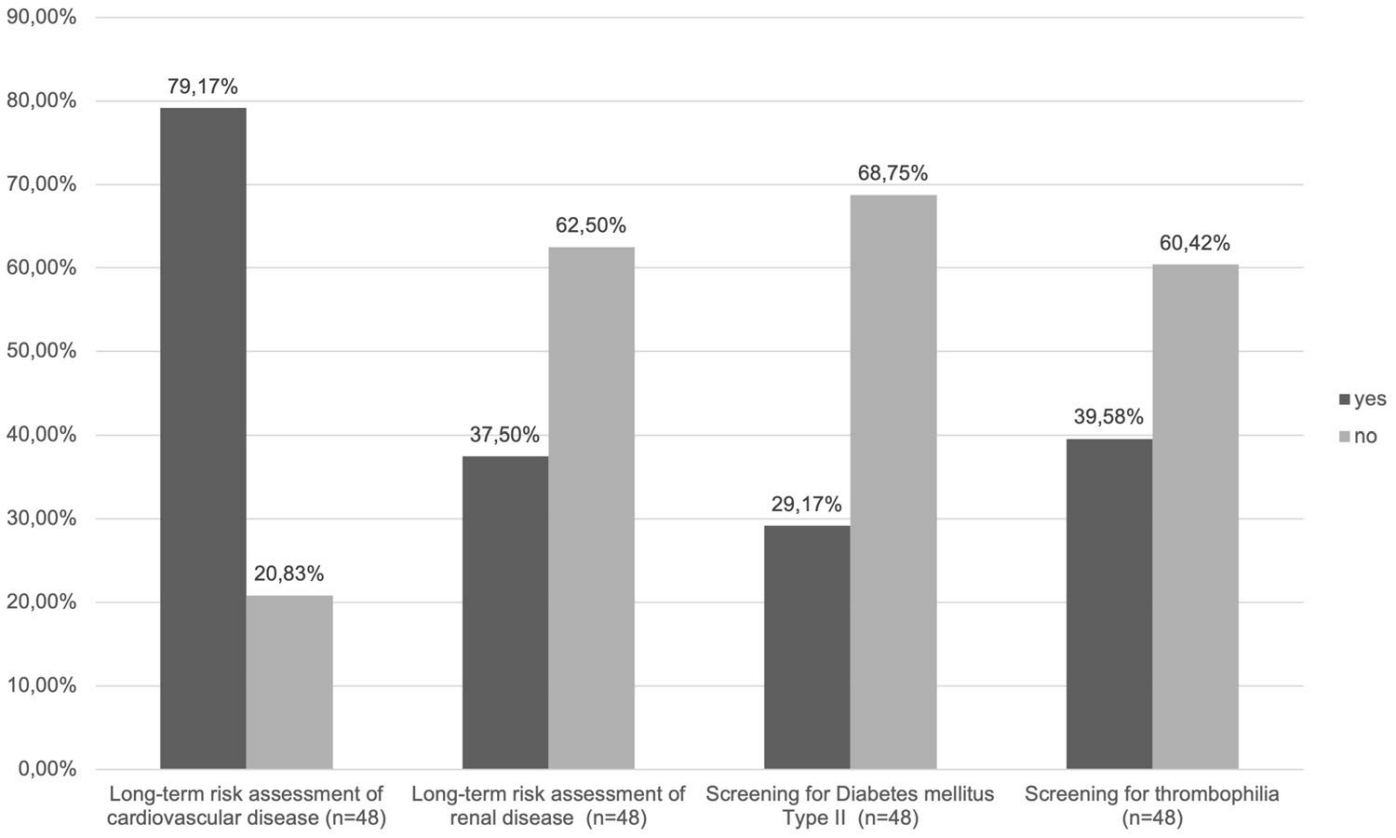
Recommendations on long-term risk counseling and diagnostics of underlying diseases. Percentages of participants recommending long-term risk assessment of cardiovascular disease, renal disease as well as screening for Diabetes mellitus Type II and screening for thrombophilia, as presented in Table 1. The *x*-axis shows (1) long-term risk assessment of cardiovascular disease (2) long-term risk assessment of renal disease (3) screening for Diabetes mellitus Type II (4) Screening for thrombophilia. The *y*-axis represents the percentage of participants answering yes/no (where *n* = 48 in all cases)

Our survey showed that 38 centers (79.17%) claimed to inform patients about future cardiovascular risks, while ten participants (20.83%) stated that patients after PE or HELLP syndrome are not counselled on this topic at their department (see Fig. 1, Table S2). Only 18 departments (37.5%) claimed to conduct consultation on long-term risk of renal disease (see Fig. 1). Regarding the timing of assessment of renal function, 10 of the participants that stated to consult on this issue suggested assessment at 3 months postpartum (20.83%), 5 recommended assessment at 6 weeks postpartum (10.42%), one participant recommended assessment at 3–6 months after delivery (2.08%), one at 6 weeks and 3 months (2.08%) and one recommended lifetime follow-up for the patient and for the child up until the age of 18 (see Table S2).

### Screening for underlying diseases

Of all 48 centers, 29 (60.42%) negated a recommendation for thrombophilia screening while 19 participants (39.6%) recommended it, as shown in Table S2. Of those recommending thrombophilia diagnostics in their follow-up protocol, 8 recommended assessment in all cases of PE or HELLP syndrome (16.67%), while 5 (10.42%) recommended assessment only in severe cases. 14 Centers (29.17%) stated to recommend diagnostics to exclude Type II Diabetes mellitus after PE or HELLP syndrome, of which 8 (16.67%) recommended screening in every case and 5 (10.42%) recommended assessment only in severe cases (see Fig. 1, Table S2). 33 departments (68.75%) stated that follow-up recommendations do not include screening for diabetes mellitus at their department.

### Risk of recurrence, consultation before further pregnancies and yearly assessments

According to the survey, all 48 of the participating obstetric departments (100%) are consulting patients on the risk of recurrence after HDP. Additionally, all of the 48 participating centers (100%) recommended conduction of a preconceptional visit before further pregnancies after HDP. See in Table 1 the recommendations on who should preferably conduct a preconceptional consultation. 14 participants gave more than one answer specifying who should conduct this consultation. On the basis of our questionnaire we determined how many of the participating centers recommend yearly assessment of blood pressure, blood glucose, blood lipids, BMI and serum creatinine (see Table 2).

**Table 1:** Consultation before further pregnancy—recommendations on who should conduct a preconceptional assessment

	*n*	%	% of the cases
Consultation before further pregnancy			
Internal medicine specialist	11	16.92	22.92%
Hospital	4	6.15	8.33%
General practitioner	2	3.08	4.17%
Obstetrician	46	70.77	95.83%
Specialist	2	3.08	4.17%
Not recommended	0	0	–
Total	65	100	–

**Table 2:** Recommendations about yearly assessment of blood pressure, blood glucose, blood lipids, BMI and serum creatinine

	Yes		No		Not mentioned	
	*n*	%	*n*	%	*n*	%
Yearly assessments						
Total (at least one recommendation)	36	75	12	25	–	–
Blood pressure	36	75	12	25	0	0%
Blood glucose	17	35.42	24	50	7	14.58%
Blood lipids	15	31.25	26	54.17	7	14.58%
BMI	19	39.58	25	52.08	4	8.33%
Serum creatinine	14	29.17	27	56.25	7	14.58%

### Life-style modification

The majority of centers (*n* = 37, 77.08%) are consulting patients after HDP on lifestyle modifications (regular exercise, healthy diet, if necessary, weight loss and smoking cessation), while 11 centers (22.92%) stated to not consult patients on this issue.

### Postpartum blood pressure monitoring

47 of 48 departments (97.92%) reported to advise patients to continue blood pressure measurements and documentation after hospital discharge. Concerning the duration of postpartum blood pressure measurements, all answers were categorized into similar groups, shown in Table 3.

## Discussion

This questionnaire-based, cross-sectional study aimed to evaluate the current recommendations of obstetricians in Austria regarding follow-up care, long-term risk counselling and risk of recurrence of HDP in future pregnancies after PE or HELLP syndrome. The basis of the survey was formed by recommendations given by three substantial guidelines on hypertensive disorders of pregnancy [21–23].

**Table 3:** Recommendations on the duration of postpartum blood pressure monitoring

	*n*	% (of all cases)	Valid %^a^
Postpartum blood pressure monitoring			
6–8 weeks	19	39.58	46.34%
3–6 months	6	12.5	14.63%
Until normal blood pressure values	7	14.58	17.07%
Depending on blood pressure values	1	2.08	2.44%
Life-long	7	14.58	17.07%
In case of antihypertensive treatment	1	2.08	2.44%
Postpartum blood pressure monitoring not recommended	1	2.08	–
Not mentioned	6	12.5	–
Total (missing answers)	7	14.58	–
Total	48	100	–

^a^Percentage of the institutions that answered the question (*n* = 41)

Our study was able to show that, according to the survey, the recommendations concerning follow-up care and risk management for patients affected by PE or HELLP syndrome, that are currently given by obstetricians in Austrian public hospitals are to a reasonable extent in line with current guidelines. Most specialists seem to be aware of and counsel patients on the association between PE and increased risk of long-term cardiovascular disease as well as the risk of recurrence. Regarding the evaluation of underlying chronic diseases such as thrombophilia or renal disease, as well as counselling on the future risk of renal disease there seems to be room for improvement according to our data. However, the overall long-term risk counselling after PE and HELLP syndrome has become well established in the hospital sector. The rising awareness in this area is certainly a consequence of numerous new insights on persistent long-term cardiac alterations and increased lifetime risk of essential hypertension, cardiovascular disease and stroke after PE [2, 24–27]. Various large observational studies have shown that follow-up and early interventions, including lifestyle modifications and patient education, are instrumental in risk reduction especially of CVD in women after pregnancy complicated by PE [28, 29]. To date, there is little knowledge on how these patients are currently managed postnatally. A recent qualitative study by Dijkhuis et al. exploring current opinions concerning follow-up for cardiovascular risks in Dutch women with a history of PE showed that follow-up has not been implemented in the north of the Netherlands [28]. Medical specialists were aware of the associated long-term risks of PE, however, the information provided to patients was minimal and inconsistent. This was generally not in line with our findings, as we were able to show that Austrian obstetricians gave a fairly high number of recommendations on risk management and follow-up care to patients after HDP concerning long-term risk of CVD, risk of recurrence and lifestyle modification. However, our observations showed a lack of counselling regarding the long-term risk of renal disease and assessment of underlying diseases such as thrombophilia. Bick et al. [16] conducted a study in the United Kingdom in which they interviewed a group of clinicians on their views and experiences on postnatal management of women who had HDP and their awareness of relevant NICE guideline recommendations [22, 30] on this issue. Their analysis highlighted several barriers in the implementation of the guidelines including poor compliance and lack of awareness of patients as well as lack of postnatal care plans. According to our data, however, physicians are updated on current guidelines on follow-up care for women with a history of PE and these seem to be reasonably implemented in clinical routine. The importance of counselling on lifestyle modifications, specifically weight management is also stressed, which was in line with the recommendations given by clinicians in Austrian obstetric departments. Melchiorre et al. [14] presented reasonable evidence to support the implementation of screening for cardiovascular disease as early as 1 year after delivery. Recommendations on the exact timing of follow-up of CVD as well as specified prevention strategies, however, have not yet been included in current guidelines and were not subject to our survey.

Despite the rising awareness of long-term risks of HDP, data on actual postpartum risk management and follow-up care after HDP are scarce. This study is the first to explore recommendations on follow-up care after PE in Austria. It, however, evaluated the awareness and recommendations of obstetric departments in public hospitals only, not investigating follow-up care in an outpatient setting, in the private sector or at general practitioners (GP). Considering the fact that patients are frequently referred to an outpatient setting or their GP for further care, an evaluation of follow-up care in this sector would be of high value. Our study had some limitations. The number of participants was rather small as the number of public obstetric departments in Austria (69) is limited. Nevertheless, in consideration of the percentage of participating clinics (69.6%), we believe that our survey is representative for the current follow-up care and long-term risk counselling after HDP in Austrian public obstetric departments. In consideration of the study design, one cannot completely exclude that respondents may have been influenced by the tendency to agree with the questions (acquiescence bias) or answered questions to fit the interpretation of the survey (demand characteristics).

Postpartum care of women after a pregnancy complicated by PE or HELLP syndrome continues to be an area of low priority and resources. Future studies should investigate the impact of timely preventive intervention in improving cardiovascular health in this group of young women at risk. Furthermore, the implementation of structured screening for cardiovascular and renal disease as well as screening for underlying diseases after a pregnancy complicated by HDP should be promoted. Recommendations on the exact timing of follow-up as well as specified prevention strategies should be included in international and national guidelines. Additionally, more detailed education of women affected by HDP about later-life health risks and involvement in decisions about ongoing care should be encouraged as it could increase compliance and in consequence improve their outcome. More in depth research, especially in the outpatient sector is warranted to substantiate our findings and to implement standardized postnatal services in the hospital sector as well as in an outpatient setting. To reduce the discussed long-term risks as well as the risk of recurrence and to increase life expectancy for patients after a pregnancy affected by HDP, adequate counselling and follow-up care on the basis of evidence-based guidelines is indispensable in an outpatient setting.

## Conclusion

Our study was able to show that postpartum counselling conducted by obstetricians concerning risk management after HDP has been largely established in the public hospital sector, however, showing deficits in follow-up care regarding the evaluation of underlying chronic diseases such as thrombophilia or renal disease, as well as counselling on the future risk of renal disease. Furthermore, our research highlights that exact recommendations concerning timing and strategies of follow-up after PE, as well as structured screening especially for cardiovascular diseases, need to be included in international and national guidelines and implemented into clinical routine to establish adequate follow-up care and improve long-term cardiovascular outcome for this young high-risk population. Additionally, evaluation of follow-up care and long-term risk counselling after PE and HELLP syndrome in the outpatient and private sector is necessary to support our findings and improve the implementation of postnatal services concerning follow-up care and risk management.

## Supplementary Information

Below is the link to the electronic supplementary material.

Supplementary file1 (tif 588 KB)
Table S1: Comparison of recommendations published by the NICE, ACOG and AWMF guidelines [18-20] with regard to questions 5 to 17 of the survey.

Supplementary file1 (tif 375 KB)
Table S2. Summary of recommendations given on long-term risk counseling and diagnostics of underlying diseases.

## Abbreviations

CVD: Cardiovascular disease
HDP: Hypertensive disorders or pregnancy
HELLP syndrome: Hemolysis, elevated liver enzymes and low platelets syndrome
PE: Preeclampsia
