# Supplementary figures and images for: Awareness of obstetricians for long-term risks in women with a history of preeclampsia or HELLP syndrome
Source: Arch Gynecol Obstet. 2021 Aug 18;305(3):581–7. doi: 10.1007/s00404-021-06181-w (PMC8918160; doi:10.1007/s00404-021-06181-w)

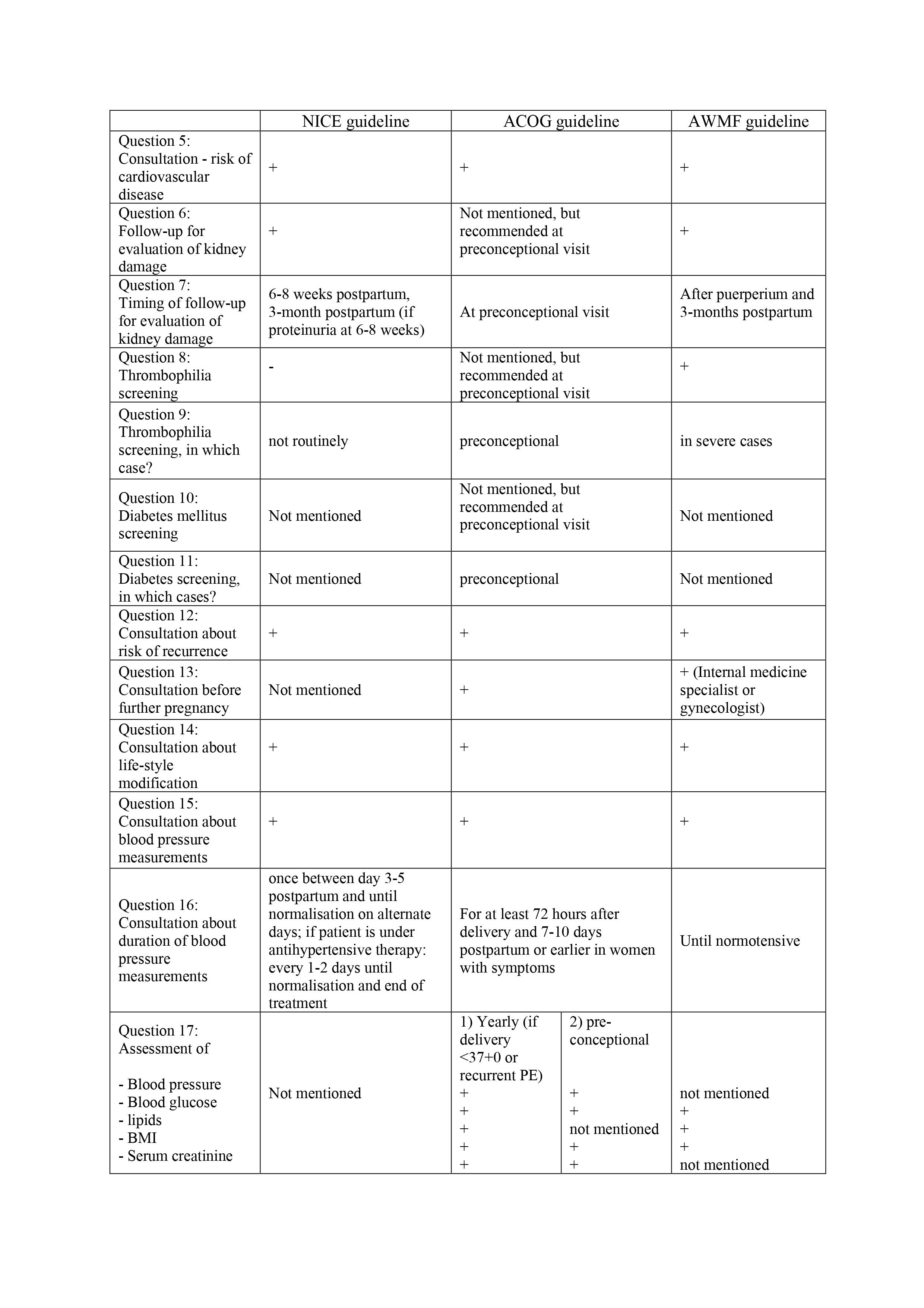

Supplement: Supplementary file 1 — Supplementary file1 (tif 588 KB) Table S1: Comparison of recommendations published by the NICE, ACOG and AWMF guidelines [18-20] with regard to questions 5 to 17 of the survey. [file 404_2021_6181_MOESM1_ESM.jpg]

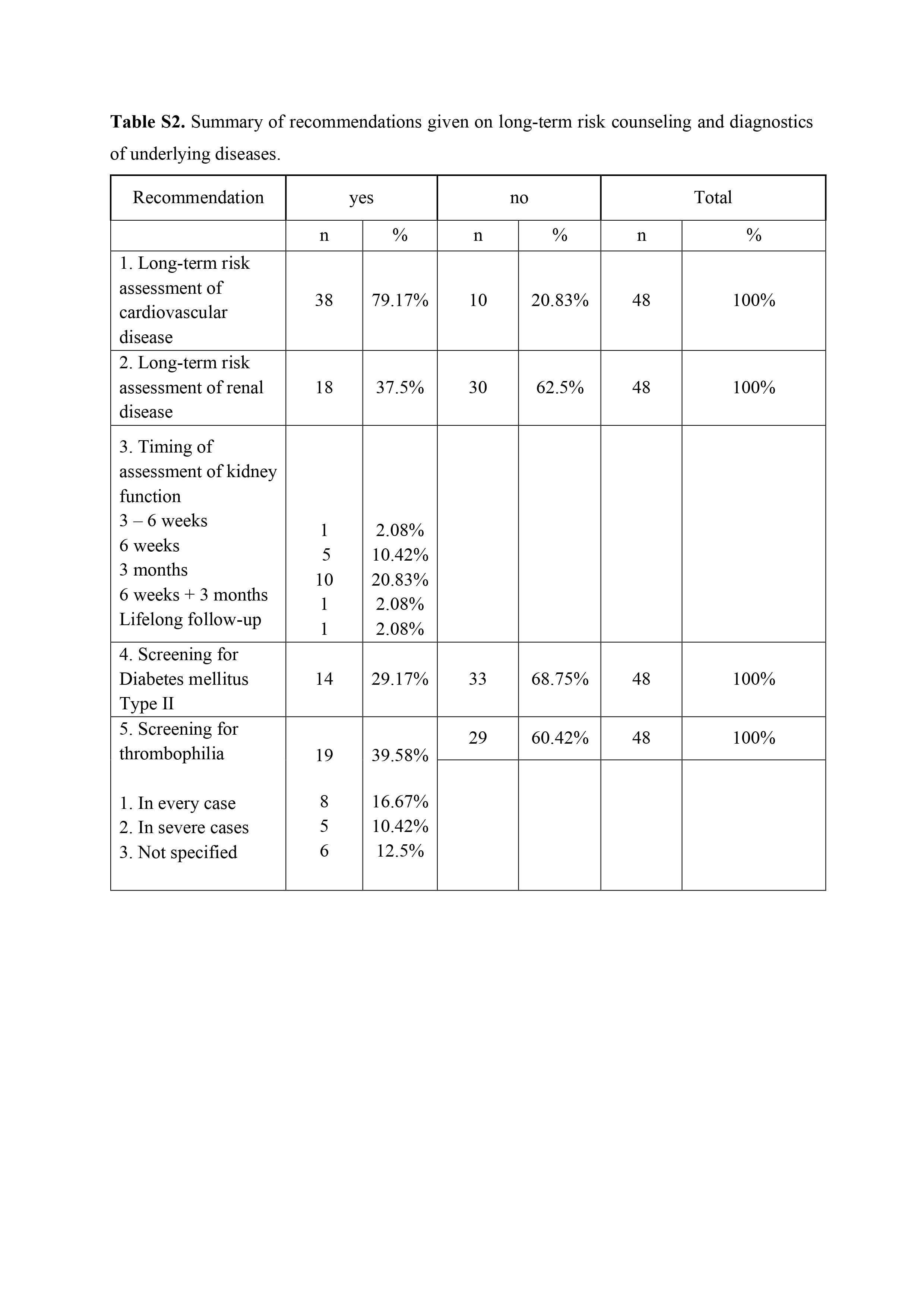

Supplement: Supplementary file 2 — Supplementary file1 (tif 375 KB) Table S2. Summary of recommendations given on long-term risk counseling and diagnostics of underlying diseases. [file 404_2021_6181_MOESM2_ESM.jpg]
